# Biocomposites of Cellulose Isolated from Coffee Processing By-Products and Incorporation in Poly(Butylene Adipate-Co-Terephthalate) (PBAT) Matrix: An Overview

**DOI:** 10.3390/polym16030314

**Published:** 2024-01-24

**Authors:** Fernanda Fabbri Gondim, João Gabriel Passos Rodrigues, Vinicius Oliveira Aguiar, Maria de Fátima Vieira Marques, Sergio Neves Monteiro

**Affiliations:** 1 Instituto de Macromoléculas Professora Eloisa Mano, Universidade Federal do Rio de Janeiro (IMA/UFRJ), Technology Center, Bloco J, Lab. J-122, Ilha do Fundão, Avenida Horácio Macedo 2030, Rio de Janeiro 21941-598, Brazil; fgondim@ima.ufrj.br (F.F.G.); jgprodrigues@ima.ufrj.br (J.G.P.R.); nviny_815@hotmail.com (V.O.A.); 2 Department of Materials Science, Military Institute of Engineering—IME, Praça General Tibúrcio 80, Urca, Rio de Janeiro 22290-270, Brazil; snevesmonteiro@gmail.com

**Keywords:** coffee industry, agro-industrial by-products, cellulose isolation, PBAT

## Abstract

With its extensive production and consumption, the coffee industry generates significant amounts of lignocellulosic waste. This waste, primarily comprising coffee biomasses, is a potential source of cellulose. This cellulose can be extracted and utilized as a reinforcing agent in various biocomposites with polymer matrices, thereby creating high-value products. One such biodegradable polymer, Poly(butylene adipate-co-terephthalate) (PBAT), is notable for its properties that are comparable with low-density polyethylene, making it an excellent candidate for packaging applications. However, the wider adoption of PBAT is hindered by its relatively high cost and lower thermomechanical properties compared with conventional, non-biodegradable polymers. By reinforcing PBAT-based biocomposites with cellulose, it is possible to enhance their thermomechanical strength, as well as improve their water vapor and oxygen barrier capabilities, surpassing those of pure PBAT. Consequently, this study aims to provide a comprehensive review of the latest processing techniques for deriving cellulose from the coffee industry’s lignocellulosic by-products and other coffee-related agro-industrial wastes. It also focuses on the preparation and characterization of cellulose-reinforced PBAT biocomposites.

## 1. Introduction

Coffee is one of the world’s most traded commodities, and its beverage is the most consumed worldwide. According to the International Coffee Organization (ICO), global coffee consumption in October 2022/September 2023 was more than 178.5 million 60 kg bags [1]. There are over 80 coffee species worldwide, but only two are commercially exploited for producing coffee beverages: *Coffea arabica* (Arabica) and *Coffea canephora* (Robusta). These two species account for 75% and 25% of the world’s coffee production, respectively. The cultivation of coffee beans takes place in tropical and subtropical regions. Brazil is the global leader in coffee production, followed by countries such as Vietnam, Colombia, and Indonesia [2,3,4].

As a result of high coffee production, coffee industry processing generates large amounts of lignocellulosic wastes rich in high organic content, such as caffeine, tannins, and polyphenols. Coffee waste disposals can contaminate water and soil, disrupting soil microbiota, so the proper management of coffee waste is required. Coffee processing wastes can be treated and incorporated into the production of value-added bioproducts, thus contributing to the circular economy and sustainable development [5,6,7].

Parchment, coffee silverskin, spent coffee grounds, and green coffee press cake can be highlighted among coffee processing by-products considered as wastes. Parchment is a fibrous covering of the coffee bean semispheres. Coffee silverskin is a by-product of the roasting process, consisting of another thin tegument that covers the coffee beans. Industry data reveal that 8 tons of roasted coffee produce about 60 kg of silverskin. Spent coffee grounds are the coffee powder residue obtained after coffee beverage preparation. Since more than half of the total coffee production is for beverage preparation, 6 million tons of spent coffee grounds are generated yearly. Finally, green coffee press cake is the by-product of green coffee beans pressed for essential oil extraction [8,9,10,11].

Several studies have investigated the use of coffee-processing by-products in different applications, for example, as an energy source based on waste burning or bioenergy (biodiesel and ethanol production) [12,13], in fermentation processes [14,15], in the production of coffee-flavored beverages [16,17], or the cultivation of microorganisms [18,19]. In addition, there are also studies involving the manufacture of food additives for human or animal feed [20,21] and even the extraction of bioactive compounds to be applied in the pharmaceutical, cosmetic, and nutraceutical fields [22,23,24].

All coffee processing by-products comprise cellulose, a natural semicrystalline polymer and one of the most abundant polymers on earth. It presents properties of low density, biodegradability, non-toxicity, high strength and flexibility [25]. Together with hemicellulose and lignin, cellulose forms one of the main structural components of plant cell walls. Because of this, coffee residues and wastes are interesting alternative sources for cellulose isolation, but few have been explored. Depending on the applied isolation method, coffee residues and wastes can lead to cellulose crystals or fibers that can be incorporated as reinforcing fillers to manufacture polymer composites, especially in packaging applications [26,27,28].

Poly(butylene adipate-co-terephthalate) (PBAT) is an aliphatic-aromatic copolyester and a synthetic polymer based on fossil resources but is biodegradable. It presents good processability and flexibility and can be applied in flexible packaging because of its characteristics. However, its high cost and lower thermomechanical properties compared with non-biodegradable polymers limit its use [29]. PBAT composites reinforced with cellulose can enhance desired properties, such as the thermo-mechanical and barrier resistance, and reduce the material’s final cost [30,31]. In addition, to improve functional properties, other fillers, such as silicate, clays or titanium dioxide, can be incorporated in combination with cellulose to provide an antimicrobial effect [32,33].

This paper aims to present a state-of-the-art review of the cellulose isolation methods applied to coffee processing wastes. It also presents works investigating the manufacture of PBAT/cellulose composites with potential packaging applications.

## 2. Coffee Processing By-Products

**Figure 1:**
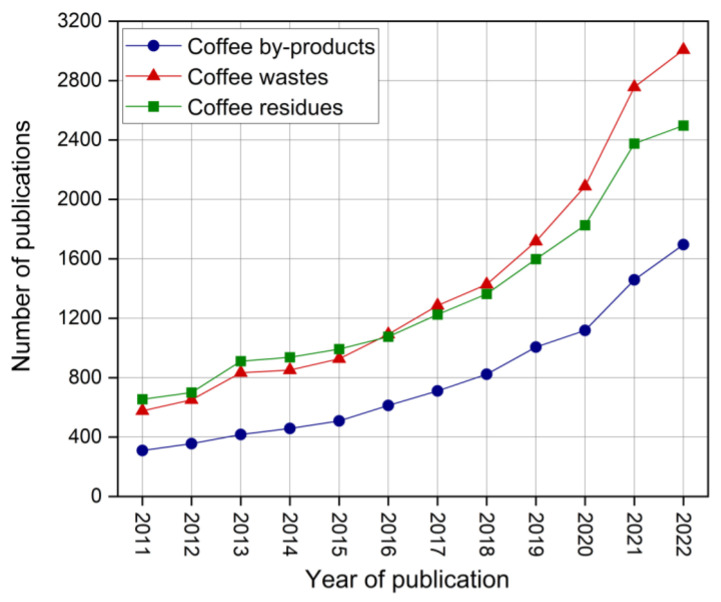
Bibliometric study of coffee by-products, wastes, and residues.

Due to the high production of coffee worldwide and high consumption of coffee beverages, large amounts of waste are generated from the coffee processing industry, and this waste requires proper disposal or reuse. Figure 1 shows the number of publications from 2011 to 2022 identified in the bibliometric study. The Scopus database was employed to search for the following keywords: coffee by-products, coffee wastes, and coffee residues. It is possible to observe an upward trend in the number of publications related to by-products, wastes, and residues from the coffee industry. However, the total number of annual publications is still relatively low, indicating a great opportunity for innovation in this area.

**Figure 2:**
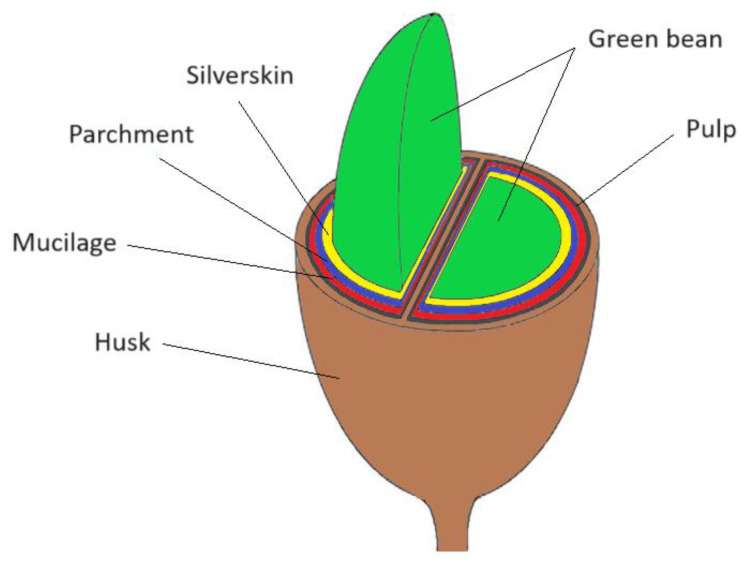
Schematic section of a coffee cherry. Source: Adapted from [35].

Coffee cherries, the fruits of coffee plants, consist of the husk, pulp, mucilage, parchment, silverskin and green coffee bean mesocarp [3,34]. After the harvest, coffee cherries pass through processing methods, which can be with or without the presence of water. Initially, the coffee fruits are de-pulped and dried, separating the beans from the other parts of the fruit. Regardless of the processing method, all the cherry’s constituents, except green beans, can be classified as solid waste [2,34]. Figure 2 illustrates a coffee cherry section (ripe fruit), indicating each part, from the inner green bean to the outer husk.

Coffee husks contain cellulose and hemicellulose, comprising about 43% and 7%, respectively, by mass. The husks include tannins, chlorogenic acids, caffeine, protein, and minerals. The pulp represents about 29% of the dry mass and is rich in sugar, caffeine, chlorogenic acids, and tannins. The mucilage is composed of water, protein, and peptides. The parchment contains cellulose (about 49%), hemicellulose (up to 32%), lignin (up to 35%) and minerals. These components are considered residual wastes of the coffee processing industry. Coffee silverskin is a residue produced during the roasting process. It is a thin tegument covering the coffee bean, representing 4% of the total grain mass. It contains cellulose (24%), hemicellulose (17%), and high levels of soluble and insoluble dietary fiber (about 60%). It is also rich in potassium, calcium, magnesium, and polyphenols [2,3,34].

Spent coffee grounds are solid residue from roasted and ground coffee, a final by-product of the brewing process. The main constituents of spent coffee grounds are cellulose, hemicellulose, sugars, fats, proteins, and minerals. This residue contains about 39% hemicellulose and 12% cellulose. The green coffee press cake is a residue of the cold-pressing extraction process of coffee essential oil from unroasted green beans [4,34,36,37].

## 3. Cellulose Isolation

Cellulose is the main structural component of plant cell walls. It constitutes 35–50% of the dry mass of leaves, stems, wood, and fruits and is present in many agricultural wastes. Cellulose is formed by β (1–4) linked D-glucose units in the linear chain. It possesses low density, biodegradability, and non-toxicity [26,38,39]. Cellulose can be isolated from these biomasses by various preparation methods. Generally, a combination of chemical, mechanical or enzymatic processes is required to isolate cellulose. The morphology and degree of crystallinity depend on the lignocellulosic source, the type of cellulose isolation methods, and the applied conditions during the process [38,40].

Cellulose crystals are obtained through hydrolysis of amorphous regions, resulting in rigid rod-like highly crystalline structures with 1–100 nm diameters and lengths of about 100 nm or more. Cellulose fibers can also be obtained by using mechanical disintegration processes combined with enzymatic or chemical treatments, resulting in flexible and entangled structures with both amorphous and crystalline domains and with diameters of 3–100 nm and lengths of several micrometers, which means that they can present a high aspect ratio [26,41].

**Figure 3:**
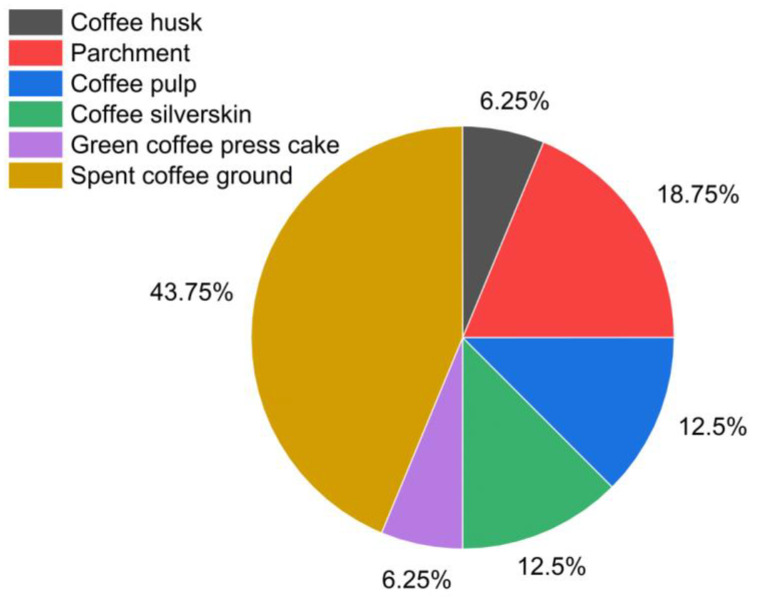
Pie chart of different types of coffee residues utilized for cellulose isolation.

Figure 3 presents a pie chart showing the percentage values of different types of coffee residues used to isolate cellulose that are investigated in this work.

Chemical treatments allow hemicellulose and lignin removal from the lignocellulosic biomass, enhancing the fibrillation process efficiency and facilitating further steps. These treatments reduce the total energy consumption required for mechanical disintegration processes [42,43,44]. The cellulose isolation processes applied to coffee residues that are reported in the literature involve the utilization of alkalis, such as sodium hydroxide (NaOH) or potassium hydroxide (KOH), as well as acidic substances, such as sulfuric acid (H_2_SO_4_), phosphoric acid (H_3_PO_4_) or acetic acid (CH_3_COOH). The use of potassium permanganate (KMnO_4_) was also reported, as well as ionic liquids, such as deep eutectic solvent (DES), and the isolation by anaerobic oxidation of primary and secondary alcohols in aldehydes and ketones using the radical 2,2,6,6-tetramethylpiperidine-1-oxyl (TEMPO) catalyst.

Mechanical disintegration techniques are also applied to the cellulose isolation of coffee residues. The disruption of the interfibrillar hydrogen bonding depends on the type of biomass, the applied level of force and the disintegration technique used [38,40]. The processes investigated in cellulose isolation were ultrasonication, steam explosion, and ball milling.

It is desirable to incorporate the isolated cellulose into a polymer matrix to manufacture functional composites, especially those applied to packaging. Due to strongly polarized cellulose not being inherently compatible with commonly used hydrophobic polymers, there is a compatibility challenge when combining polymers and cellulose. Some pretreatments may reduce the degree of hydrophilicity of cellulose due to the high reaction levels of hydroxyl groups on its surface, resulting in chemical surface modifications. However, further chemical modifications after cellulose isolation can still be required [41,42,43,44,45,46].

Table 1 shows the type of coffee residue, the type of cellulose (i.e., cellulose nanocrystals (CNCs), cellulose nanofibers (CNFs) or microfibrils), diameter, length, crystallinity index and yield of the process of the main reference studies. 

The following sections will discuss studies that have used cellulose isolation processes of lignocellulosic biomasses from the coffee processing industry to obtain cellulose.

### 3.1. Chemical Treatments Applied on Wastes from the Coffee Processing Industry

In a study by Collazo-Bigliardi et al. [47], cellulose nanocrystals (CNCs) were obtained from coffee husks by alkaline treatment using 4 wt.% NaOH solution, followed by bleaching treatment in acetate buffer solution (acetic acid, sodium chlorite and water) and acid hydrolysis with H_2_SO_4_. The chemical composition, morphology, microstructure, degree of crystallinity of CNCs and the total process yield were evaluated. The chemical composition results revealed that the cellulose content ranged between 34% and 35% for untreated coffee husks. Scanning electron microscopy (SEM) analysis showed crystal rod-like morphologies and presented an aspect ratio (L/d) of around 10. The degree of crystallinity was calculated by X-ray diffraction (XRD), and was found to be greater than 90%. Alkaline and bleaching treatment yielded 60% relative to the initial dry mass. The authors suggested that cellulose from coffee husks is an interesting source of cellulosic reinforcing material for different applications, such as packaging.

Dominici et al. [56] investigated two different treatments applied to coffee silverskin. The first was an alkaline treatment with 0.5 M NaOH solution, stirred at 80 °C for 2 h. A 0.1 M HCl solution was then added to complete neutralization, and then the treated fiber was washed several times to remove the reaction products (NaCl salts). The second method was an alkaline treatment under the same conditions, followed by an esterification process with palmitoyl chloride to achieve hydrophobization of the coffee silverskin. Fourier transform infrared spectroscopy (FTIR) examination of the treated coffee silverskin showed an absence of bands at 1742 cm^−1^ and 1247 cm^−1^, which corresponds to acetyl and uronic ester groups of hemicelluloses and C–O stretching of acetyl groups of lignin, respectively, demonstrating that the chemical treatments were able to remove noncellulosic components. A thermogravimetric analysis (TGA) also showed a slight reduction in the thermal stability of the esterified coffee silverskin fibers, which could be attributed to the limited stability of ester bonds. However, a temperature increase in the main peak of the treated fibers suggests that they can be incorporated into a biobased polyethylene matrix.

Frost and Foster [53] isolated CNCs from spent coffee grounds. First, a soxhlet extraction was performed with ethanol and toluene to remove lipids. Then, a bath containing NaOH solution was used to remove the soluble hemicellulose. A solution of hydrogen peroxide and acetic acid was also used to remove the remaining noncellulosic components. Finally, acid hydrolysis using H_3_PO_4_ was performed. The yield of CNCs was about 10%, which was expected due to the low cellulose content in spent coffee grounds (8–10% by mass). CNCs obtained by hydrolysis with H_3_PO_4_ showed greater thermal stability than those CNCs obtained by conventional routes using H_2_SO_4_. The degree of crystallinity of CNCs was found to be 70%, in accordance with the literature. The authors suggested the incorporation of the obtained CNCs as reinforcement fillers in nanocomposites to be applied in the polymer engineering field.

Kanai et al. [54] prepared CNFs from spent coffee grounds by performing TEMPO-mediated oxidation. TEMPO-oxidized cellulose nanofibers (TOCNFs) were compared with microcrystalline cellulose (MCC), and both were characterized using TGA and XRD analysis. The TGA results showed that the TOCNFs started to degrade earlier than MCC and also presented higher levels of char residue than MCC due to the influence of sodium cations as counterions. The XRD results showed that the degree of crystallinity of TOCNFS (72%) was slightly lower than MCC (79%). This study indicated that the produced TOCNFs were incorporated into PVA, enabling the manufacture of composites, which could contribute to the development of sustainable green chemistry.

Dutta et al. [55] used chemical treatments to obtain CNCs from coffee grounds by applying several steps. First, the authors subjected the residue to alkaline treatment, using 3 wt./vol.% of KOH solution at 90 °C for 2 h. The treated residue was washed with distilled water, neutralized with 10 wt.% of hydrochloric acid, filtered and dried. Then, it was further dispersed in water and treated with sodium chlorite and acetic acid solution. After six repetitions of this step, the residual material was treated with 17 wt./vol.% NaOH and 10 wt.% acetic acid solution. Finally, the obtained cellulose was treated with 64 wt.% of H_2_SO_4_ at 45 °C for 60 min, following which the reaction was quenched with cold water to obtain CNCs. The yield of CNCs was calculated and found to be 7%. Transmission electron microscopy (TEM) was used to determine the dimensions of the CNCs, and their length was approximately 120 nm. The obtained CNCs were explored as promising candidates for tissue engineering, especially bone tissues.

Malarat et al. [50] obtained CNCs from coffee pulp. The coffee pulp was treated with 4 wt./vol.% of NaOH solution. Then, the alkali-treated pulp was bleached with an acetate buffer solution, sodium chlorite and distilled water and then further treated via acid hydrolyzed with 65 wt.% of H_2_SO_4_. The results revealed that alkali and bleaching treatments could remove noncellulosic components, such as lignin and hemicellulose. The cellulose content of the coffee pulp was 31.2%, and after the alkaline treatment and bleaching process, its content increased to 85.8%. The crystallinity index increased from about 20% to more than 80% with the removal of the amorphous phase of cellulose. The CNCs presented a needle shape with an average diameter of 16.03 ± 4.70 nm. They were used to reinforce a polyvinyl alcohol (PVA) film via solvent casting to improve the PVA properties.

Panyamao et al. [48] isolated cellulose nanofibers (CNFs) from coffee parchment using a class of ionic liquid called deep eutectic solvent (DES). DES is a mixture of two or more components in a certain proportion with a melting point much lower than that of the individual constituents. These solvents have minimum environmental impacts with low toxicity and excellent biodegradability compared with conventional ionic liquids. Choline chloride, oxalic acid, and water in a 2:2:1 molar ratio were used as a pretreatment under microwave heating, followed by a two-step alkaline hydrogen peroxide process. The cellulose content of untreated coffee parchment was 31.9%, and the recovery yields of cellulose were maintained at over 80%. Their TGA results showed that the treated CNFs presented higher Tmax (350 °C) compared with the untreated CNFs (335 °C), with better thermal stability. The XRD results also showed an increase in the crystallinity index from 48.9% for untreated parchment to 73.4% for treated CNFs. The increase in crystallinity was attributed to destroying the amorphous structure (hemicellulose and lignin complex) in the biomass. This study points out that CNFs obtained from coffee parchment are a promising renewable nanomaterial for various biomedical and pharmaceutical applications.

Rizkiansyah et al. [51] conducted a study in which they isolated cellulose from coffee pulp using an alkali treatment method with 3% weight of sodium hydroxide (NaOH), followed by a bleaching process with hydrogen peroxide. The research focused on determining the cellulose content in various stages of the pulp: firstly, in its washed state; secondly, after undergoing alkali treatment; and finally, post-alkali treatment and subsequent bleaching. Additionally, the study assessed the morphology and crystallinity degree of the cellulose derived from these processes. The results showed that the cellulose fraction increased after alkali treatment, reaching around 71.05%, from only 12.55% in the native conditions. The SEM images showed smooth surfaces with flake-like, porous, and some helical-shaped microfibrils. The crystallinity values were 55.89% for washed coffee pulp and 59.63% for treated pulp (alkali treated, followed by bleaching treatment). The authors suggested that the nitration reaction to coffee pulp-based cellulose be applied in various applications, from plastics to solid propellants for military ammunition and rocketry.

Rodríguez et al. [49] isolated cellulose microfibrils from coffee parchment through acid hydrolysis, alkaline hydrolysis and bleaching. An aqueous solution of 2.5 and 5% (*v*/*v*) of HNO_3_ was applied by reflux heating for 60, 120, and 180 min for acid hydrolysis. After that, the coffee biomass was subjected to 1.5 and 3% (*m*/*v*) NaOH solution for 45 and 90 min, and then equal parts of NaClO and CH_3_COOH (1:1 and 2:2) were used for bleaching. The results showed that acid hydrolysis and alkali hydrolysis could break down the coffee parchment lignocellulosic compounds, allowing the solubilization of lignin and hemicellulose. This was corroborated by FTIR results, with a decrease in the intensity of the bands corresponding to ketone, acid, and ester groups of hemicellulose and lignin, and the aryl–alkyl ethers of lignin.

### 3.2. Mechanical Disintegration Techniques Applied to Wastes from the Coffee Processing Industry

In the steam explosion process, the swollen cellulosic fiber mass is exposed to high-pressure steam for short periods. Elevated pressure and temperature, followed by the sudden release of pressure, generate shear forces that defibrate the material, resulting in the formation of microfibrils. This technique has a lower environmental impact since no hazardous chemicals are used. There is also a lower energy consumption compared with other mechanical processes [57,58,59]. Ravindran et al. [60] applied a two-step sequential pretreatment to achieve enhanced lignin removal on spent coffee grounds. The authors subjected the residue to a concentrated pretreatment, using H_3_PO_4_ and acetone (C_3_H_6_O), followed by a steam explosion treatment with ammonia solution, using high pressure (15 lbs/sq inch) and high temperature (120 °C) for 30 min. Their SEM images showed a reduction in size for pretreated spent coffee grounds, which was attributed to the removal of hemicellulose structures. The FTIR results also showed that peaks characteristic of lignin, such as 1509, 1464, and 1422 cm^−1^, were absent in the spectrum, confirming the efficiency of the treatments.

Brito et al. [11] applied the steam explosion method to partially remove the amorphous components of green coffee press cake. The steam explosion was performed at a temperature of 180 °C and pressure of 2 bar in 10 min explosion cycles. This study investigated the best retention time for green coffee press cake fibers swollen in water inside an autoclave. A total of five cycles of the steam explosion were performed. The TGA results showed that the mechanical treatment applied to the fibers resulted in a narrowing of the maximum decomposition speed DTG peak, which indicated some partial removal of the amorphous components of the fibers. After the explosion cycles, an increase in the fiber’s moisture content was observed, corresponding to an increase in its hydrophilic character since there was an increase in the exposed cellulose surface resulting from the steam explosion technique. Fibers treated for 30 min presented an increase in thermal stability. However, T_onset_ and T_15%_ decreased after the third cycle due to the possible degradation of cellulose molecules. The authors concluded, therefore, that 30 min was the optimal retention time. The resulting fiber was chosen to be further incorporated into a polymer matrix to manufacture composites.

Reis et al. [7] also used the steam explosion method to obtain microfibrillated cellulose from coffee parchment residue. An alkaline treatment was first performed using NaOH solution to remove lignin and hemicellulose, followed by steam explosion. Then, the mechanical disintegration method was conducted in hot water at 120 °C, with less than 2 bar pressure and rapid decompression. The resulting material was then subjected to high shearing forces using a homogenizer to improve defibrillation. Parchment coffee fibers, fibers after alkaline pretreatment, fibers obtained after the steam explosion, and fibers obtained after the steam explosion and high shearing forces were characterized by FTIR and TGA. The FTIR spectra of the untreated parchment showed a band at 1730 cm^−1^, which is characteristic of hemicellulose. The absence of this band in the spectra of the treated fibers demonstrated that the pretreatment efficiently removed hemicellulose from the biomass. In the TGA analysis, the treated fibers had greater thermal stability compared with untreated parchment, with a sharp increase for fibers after the steam explosion and high shearing forces. Most cellulose amorphous regions were usually destroyed during the treatments, increasing the degree of crystallinity of the materials and, consequently, their thermal stability. The cellulose content for this residue was 22%. Therefore, the recovery yield of cellulose was 20.5%.

Ultrasonication is a technique in which the cavitation process generates high- and low-intensity oscillating ultrasonic waves, forming microscopic gas bubbles that are expanded and imploded, breaking down and defibrillating cellulose fibers [61]. Sung et al. [52] isolated CNCs from coffee silverskin. This coffee by-product was ground, dried, treated with KOH solution, and then bleached with sodium hypochlorite (NaClO) solution with pH adjusted to 5 by acetic acid. Then, it was subjected to acid hydrolysis by an alkaline process and hydrolyzed with H_2_SO_4._ The colloidal suspension was dialyzed against distilled water until a neutral pH was reached. The final suspension was sonicated for 15 min in an ice bath and then freeze-dried to obtain the CNCs. A TEM analysis confirmed the size of the CNCs.

Furthermore, XRD analysis evaluated the degree of crystallinity of the CNCs, and the results showed values greater than 70%. The CNCs were produced at a total yield of 12%, indicating the high degree of removal of noncellulosic constituents. The CNCs were incorporated into a poly(lactic acid) (PLA) matrix to develop nanocomposites with enhanced barrier and mechanical properties.

Ravindran et al. [62] used KMnO_4_ and water bath ultrasonication at room temperature to pretreat spent coffee grounds. The authors investigated different exposure times to the oxidizing agent, varying the sonication time. A compositional analysis revealed that the spent coffee grounds contained 8.6% cellulose. Treatment with KMnO_4_ at a concentration of 4 wt.% followed by 20 min of ultrasound resulted in a lignin removal of 46%, attributed to the cavitation effect and oxidation of the ester and ether bonds. The water bath ultrasonicator led the generation of highly reactive species, such as hydroxyl ions, hydronium ions, and ozone, which reacted with the spent coffee grounds, contributing to the decomposition of the lignocellulosic biomass. This process was studied for application in bioethanol production. However, it can be successfully conducted on cellulose isolation when combined with other techniques to incorporate cellulose into a polymer matrix.

Ball milling is a process in which the cellulosic material is placed in a cylindrical vessel containing balls. This vessel rotates, and the centrifugal forces combined with the high-energy collision between the balls generate shear forces that promote defibrillation. In addition, the ball size and weight influence the final product and morphology [63]. Lee et al. [64] prepared particle-reinforced PVA composites from coffee wastes. First, used coffee grounds were ball-milled, and the coffee waste was extracted several times with hot water, dispersed into distilled water and then filtered using a glass filter with a mesh size of 700 nm. Then, composite films were cast to form PVA/coffee composites with 1%, 2% and 3% wt. contents. The particle size distribution of the used coffee grounds prepared via ball milling was evaluated, and the average diameter of the coffee grounds was 240 nm. The aim of this study was not cellulose isolation. However, the results showed the efficiency of this technique for particle size reduction, which can be combined with other pretreatments to produce cellulose.

Giang et al. [65] investigated a ball milling process combined with an ultrasonic liquid processor on spent coffee grounds. The residue was first water-extracted, in which it was washed with boiled distilled water to remove the water-soluble components, and dried before the ball milling process. It was milled in a cylindrical ceramic ball mill at a speed of 225 rpm for 40 min. The mixtures were transferred to a 50 mL centrifuge tube and placed in an ultrasonic liquid processor. The sonication amplitude parameter was set at 30, 50, and 70%, and the sonicated mixture was then filtered using a qualitative filter paper. The dynamic light scattering (DLS) technique showed a reduction in average size of the treated spent coffee grounds, with a sharp decrease observed in the results between the 30% (242.6 ± 12.5) and 50% (154.3 ± 1.0) amplitude setting and a further slight decrease at the 70% setting (152.3 ± 0.9). In this study, the treated spent coffee grounds were incorporated into a PVA matrix to manufacture composites for application as an ultraviolet (UV) shielding material.

## 4. Poly(butylene Adipate-Co-Terephthalate) (PBAT)

Biodegradable polymers have gained attention in the market in several areas, mainly in the packaging industry, because of their lower environmental impact and waste generation. The degradation process by microorganisms occurs under suitable conditions of humidity, temperature, and oxygen availability without generating toxic residues. Among them, PBAT stands out as a biodegradable polymer from fossil resources. It is a random copolymer prepared by a transesterification reaction and is formed from aromatic and aliphatic chains from 1,4-butanediol (BDO), adipic acid (AA), and terephthalic acid (PTA) monomers [29,66,67].

PBAT has good ductility and flexibility, with a melting temperature (T_m_) of around 120 °C and a glass transition temperature (Tg) of −17 °C. In addition, its mechanical properties are similar to those of low-density polyethylene, making PBAT an interesting polymer to be applied in packaging, such as plastic bags. However, its low tensile strength, low modulus, low transparency, water vapor permeability, and higher cost compared with other non-biodegradable synthetic polymers limit its use. In this sense, PBAT composites can have improved thermal, mechanical, and barrier properties compared with neat PBAT [68,69,70,71].

The most common polymer blends investigated in the literature are those of PBAT with poly(lactic acid) (PLA) using different ratios and compatibilizers to improve the polymer’s interface [72,73,74,75]. Other polymer blends that have been studied include PBAT with polycaprolactone (PCL) [76], poly-3-hydroxybutyrate (PHB) [77], poly(butylene succinate) (PBS) [78], poly(succinate-co-butylene adipate) (PBSA) [79] and starch [80].

Different types of fillers can be incorporated into the PBAT matrix or blends containing PBAT, such as zinc oxide (ZnO) [81], titanium dioxide (TiO_2_) [82], or silicon dioxide (SO_2_) [83]. In addition, clays [84], graphene or graphene oxide [85,86], different natural fibers [87] and cellulose, such as CNFs [88,89] and CNCs [90,91] have also been studied and are the main focus of this review. Cellulose can be combined with the mentioned fillers to confer antimicrobial properties or achieve a synergistic effect [43,92].

The main challenge of PBAT/cellulose composite manufacturing is compatibilization. When cellulose is compatibilized and well dispersed in the PBAT matrix, some properties of composites can be improved, such as the mechanical, thermal, water vapor, or gas barrier resistance properties. However, cellulose has a hydrophilic character due to hydroxyl groups on its surface, and it is often necessary to carry out chemical modifications to improve the polymer–filler interface [40,41,45].

The following sections of this work will present some studies examining the preparation and properties of PBAT composites containing cellulose.

### 4.1. PBAT Nanocomposites Containing CNCs

Morelli et al. [30] investigated the incorporation of CNCs in a PBAT matrix. The CNCs underwent chemical modifications and were grafted with an aromatic isocyanate (4-phenyl butyl) and an aliphatic one (octadecyl). PBAT nanocomposites containing 5 and 10 wt.% of CNCs, either modified with octadecyl isocyanate (CNCoct), modified with 4-phenyl butyl isocyanate (CNCpb) or unmodified (CNCref), were prepared by solvent casting in chloroform solution. Chemical modifications decreased the hydrophilic character of the CNCs, improving the dispersion and distribution in the polymer matrix. Raman spectroscopy results indicated that the CNCs modified with a 4-phenyl butyl compatibilizer agent presented good interaction due to π-π between phenyl rings grafted into CNCs and the polymer chain’s aromatic rings of terephthalic acid, resulting in significant improvement in the mechanical properties of nanocomposites containing CNCpb. All the nanocomposites showed increased modulus and tensile strength compared with neat PBAT. However, the nanocomposite containing 10 wt.% CNCpb showed 121% and 40% increases in the modulus and yield stress, respectively. Cellulose hydrogen bond interactions were not completely avoided. Therefore, at levels above 7 wt.% of CNCs, composites presented a state of percolation, contributing to improved mechanical properties.

Zhang et al. [93] incorporated CNCs and acetylated CNCs modified by acetic anhydride (ACNCs) into a PBAT matrix through the melting mix method. Composites containing 0.5 and 2.0 wt.% of CNCs and ACNCs were prepared to compare their rheological behavior. The PBAT/ACNC composites presented higher elastic modulus, loss modulus and complex viscosity than the PBAT/CNCs. The moduli of PBAT/ACNC composites increased with increasing ACNC content, indicating an enhanced interfacial adhesion between CNCs and the PBAT matrix, which would contribute to the mechanical properties of the PBAT/ACNC composites. Dynamic mechanical analysis (DMA) was also performed, and the PBAT/ACNC composites presented higher storage moduli than the PBAT and PBAT/CNC composites; furthermore, the elastic modulus and tan d of the PBAT/ACNC composites remained at a high level over a wide temperature range, indicating enhanced rigidity of the amorphous phase and a decrease in the chain mobility of the PBAT/ACNC composites.

Pinheiro et al. [94] studied the effects of CNCs modified with octadecyl isocyanate on the mechanical properties of PBAT nanocomposites. The nanocomposites were prepared by melt fusion, with CNC contents of 3, 5 and 7 wt.%. The chemically modified CNCs exhibited higher crystallinity (increment of 6.6%) and improved thermal stability (increment of 52 °C on T_max_), as shown by XRD and TGA results. Also, grafted CNCs showed increased compatibility and improved the adhesion between modified CNCs and the polymer matrix. This leads to efficient load transfer and an improvement in mechanical properties. The modulus of the samples containing 5 and 7 wt.% of modified CNCs was about 12 and 42% higher, respectively, than the values for the neat PBAT.

Silva et al. (Silva et al., 2020) [95] prepared and characterized PBAT and thermoplastic starch (TPS) (from cassava) blends reinforced with CNCs isolated from eucalyptus pulp by acid hydrolysis. PBAT/TPS blends containing 2, 4, and 6 wt.% of CNCs (also containing glycerol, citric acid, and stearic acid) were prepared by extrusion. The thermal and mechanical properties of the samples were analyzed. The results showed that including CNCs modified the mechanical and thermal behavior of the PBAT/TPS blends. There was an improvement in mechanical properties, especially for the PBAT/TPS blend containing 4 wt.% of CNCs. Tensile tests showed that this sample had a 120% increase in Young Modulus (E), a 46% increase in yield point (σ) and a 44% increase in the elongation at break (ε). Furthermore, the Tg values, obtained by dynamic-mechanical analyses (DMTA) and differential scanning calorimetry (DSC). also increased. This indicates a good dispersion between blend interphases, consistent with the images obtained from the SEM analysis.

Vatansever et al. [96] developed PBAT nanocomposites containing 1, 3 and 5 wt.% of CNCs. The nanocomposites were prepared by solution casting, using dimethylformamide (DMF) as a solvent and fusion by melting. For melt processing, masterbatches containing 7 wt.% by mass of CNCs were prepared, mixed in solution with DMF and then with PBAT pellets and processed in a double-screw extruder. The nanocomposites were characterized by morphology, degree of crystallinity, and oxygen permeability properties. TEM analysis verified that the nanocomposites prepared by solution casting presented a better distribution of CNCs in the polymer matrix than the composites prepared by extrusion. Small agglomerates were identified for the composition containing 5 wt.% of CNCs prepared by fusion. This was due to masterbatch dilution, which generated a stimulus to reagglomeration, increasing the CNCs’ mobility in the melted composite under a high shear rate. Incorporating CNCs considerably decreased gas permeability, especially in the samples processed by solution casting, as shown by oxygen permeability (OTR) analysis. The sample containing 5 wt.% of CNCs had a decrease in permeability of 41% due to tortuous pathways created during the gas passage. The degree of crystallinity, as observed by DSC and XRD, did not significantly change with the incorporation of CNCs.

Arslan et al. [97] prepared PBAT nanocomposites containing 3 wt.% of unmodified CNCs and chemically modified CNCs (mCNCs). The chemical modification was performed by grafting poly(glycidyl methacrylate) (PGMA) using three different contents of GMA monomers. Nanocomposites were prepared using an internal melt mixer. Melt rheological analysis was performed, and the results showed increased complex viscosity and storage modulus when mCNCs were added at low frequencies. This was attributed to the reinforcing effect of well-dispersed mCNCs and the enhancement of interfacial interactions between mCNCs and PBAT through epoxide groups of PGMA and the carboxylic acid chain ends of PBAT.

Dhali et al. [98] developed PBAT nanocomposites containing silane-modified CNCs, functionalized by triethoxyvinylsilane (TEVS) and trimethoxyphenylsilane (TMPS). Nanocomposites of PBAT with 3 wt.% of CNCs modified with TMPS, 5 wt.% modified either with TEVS or TMPS, and 5 wt.% of unmodified CNCs were prepared individually by melt processing. Water vapor permeability tests (WVTR) and oxygen transmission rate tests (OTR) were performed on the nanocomposites. The WVTR results showed that incorporating unmodified and modified CNCs reduced the transmission rate from 70.4 g m^−2^ day^−1^ for neat PBAT to 6.9 g m^−2^ day^−1^ for the nanocomposite containing 5 wt.% of unmodified CNCs. The WVTR values significantly decreased for the nanocomposite containing 5 wt.% of CNCs modified with TMPS, which presented the lowest WVTR value of 38.3 g m^−2^ day^−1^. This could be attributed to hydrophobic silane groups that restricted the diffusion of water molecules and created an extensive tortuous path due to the dispersion of modified CNCs. The OTR results were also significantly reduced, from 1057.6 cc m^−2^ day^−1^ for neat PBAT to 588 and 496 cc m^−2^ day^−1^ for nanocomposites containing 5 wt.% of CNCs modified with TEVS and TMPS, respectively.

### 4.2. PBAT Composites Containing CNFs

Mukherjee et al. [99] studied CNF dispersion in the PBAT matrix. First, neat polymer and nanocomposites containing 0.2, 0.5, 0.75 and 1 wt.% of CNFs were processed in a mixer and pelletized. Subsequently, the samples were injected, molded, and characterized by TEM and DSC analysis. Nanocomposites containing up to 0.2% wt.% presented a good dispersion of CNFs, but higher cellulose contents resulted in the formation of agglomerates. Decreases in the composites’ Tg values were observed through TGA analysis, with a pronounced decrease in the nanocomposite containing 0.2 wt.% CNFs, which indicated the mobility restriction of polymer chains in this composition, corroborating the results obtained from the TEM analysis. In addition, the degree of crystallinity of the nanocomposites increased with the increase in CNF content, showing that CNFs acted as nucleating agents.

Edlund et al. [100] chemically modified the surface of CNFs using a polymer coating synthesized by admicellar polymerization (ADPM). The cationic surfactant hexadecyl pyridium chloride monohydrate (CTP), hexyl acrylate monomer and water were mixed to form a thin polyacrylate layer on the surface of the CNFs. Nanocomposites containing 5 and 20 wt.% of modified and unmodified CNFs were prepared in a double-screw extruder. Field emission scanning electron microscopy (FE-SEM) and tensile tests were performed. The FE-SEM images showed that chemical modification resulted in a reduction in entanglements. In the tensile tests, there was no significant improvement in the mechanical properties of composites with modified CNFs compared with those containing unmodified CNFs, except for the higher elongation at break for the composition containing 5 wt.% of CNFs.

Lai et al. [101] developed nanocomposites containing epoxide-modified nanosilica (SiO_2_-EO) and amine-treated cellulose nanofiber (CNF-NH_2_). The study compared nanocomposites that included a combination of both SiO_2_-EO and CNF-NH_2_ with nanocomposites containing solely SiO_2_-EO, CNF-NH_2_ or neat PBAT. The researchers observed that incorporating both SiO_2_-EO and CNF-NH_2_ into the PBAT matrix led to a synergistic improvement in mechanical properties. This enhancement was attributed to the chemical interactions between the modified nanoinclusions, suggesting that the combination of these two materials within the polymer matrix plays a crucial role in enhancing the overall performance of the nanocomposites. The composite containing 0.6 vol. % of SiO_2_-EO and 0.8 vol. % of CNF-NH_2_ within the PBAT showed an increase in Young’s modulus of 47% compared with neat PBAT, with a maximum increase of 16% for 0.6 vol. % of SiO_2_-EO alone and 12% for 0.8 vol. % of CNF-NH_2_ alone. The SEM results showed good dispersion and the formation of a reinforcing network microstructure throughout the nanocomposites.

Montero et al. [102] prepared PBAT nanocomposites containing CNFs embedded in cinnamon essential oil (EO) by wire extension. The modified CNFs were impregnated with cinnamon essential oil (CEO) nanocomposites with modified and unmodified CNFs prepared by mixing with PBAT solution at concentrations of 0.5%, 1%, and 3%, using a high-speed homogenizer. The nanocomposites were analyzed by TGA and were also evaluated as packaging for strawberries. The TGA results showed that incorporating unmodified CNFs increased the T_onset_ values by 4 °C due to good dispersion. On the other hand, the T_onset_ values for nanocomposites with modified CNFs decreased by 10 °C, possibly due to the essential oil volatilization, which could accelerate the thermal degradation. The nanocomposites containing 5.0 wt.% of modified CNFs presented the highest oil release, resulting in lower strawberry weight loss after 15 days of storage, better freshness preservation, and no fungal attack.

## 5. Conclusions

The coffee industry generates large amounts of processing by-products, wastes and residues, such as coffee husk, parchment, silverskin, spent coffee grounds, and green coffee press cake. If discarded in the environment, these wastes and residues can contaminate water and soil. Therefore, one of the alternatives for their disposal is cellulose isolation, which is applied through a combination of chemical and mechanical methods to a lignocellulosic biomass so that cellulose can be obtained in the form of crystals or fibers. These can be further incorporated as reinforcing fillers into a polymer matrix, especially biodegradable ones.

Poly(butylene adipate-co-terephthalate) (PBAT) is a biodegradable polymer that should be highlighted, especially in packaging applications, due to its mechanical properties, which are similar to those of low-density polyethylene. However, its high cost and thermomechanical properties are inferior to those of other non-biodegradable polymers, limiting its use.

The studies outlined in this review demonstrate that it is possible to obtain PBAT/cellulose nanocomposites that present improved thermomechanical and barrier properties compared with neat PBAT. However, there is a need to investigate different combinations of cellulose isolation processes on coffee residues, wastes and by-products that can contribute to different morphologies, sizes and degrees of crystallinity, factors which directly impact the final nanocomposite properties. In addition, chemical modifications performed on cellulose surfaces can be a good alternative to enhance the polymer–filler interface, contributing to enhancements in general composite properties. Finally, it is suggested that metal oxides, clays, graphene or graphene oxide could also be incorporated with cellulose to promote a synergistic effect on the final material properties.

In short, the following conclusions can be highlighted:

- Isolating cellulose from coffee residues and wastes is relatively underexplored but presents a promising avenue for sustainable development. This approach not only offers a method to recycle and repurpose coffee industry by-products but also contributes to the creation of environmentally friendly materials and products. By harnessing the potential of these waste materials, we can move towards more sustainable practices in both waste management and material production;
- PBAT/cellulose composites can present improved properties compared with neat PBAT, especially when combined with other fillers, and can be applied to the manufacturing of packaging;
- Some challenges remain to be overcome, such as finding a combination of processes that presents less environmental impact and results in desirable cellulose morphologies, especially regarding nanometric size. It is also necessary to enhance polymer–filler compatibilization through cellulose surface modifications.

## Figures and Tables

**Table 1:** Correlations between the type of coffee residue, type of cellulose, diameter, length, crystallinity index and yield of the process of the main studies involving isolation of cellulose from coffee residues.

Type of Coffee Residue	Type of Cellulose	Diameter (nm)	Length (nm)	Crystallinity Index (%)	Yield (%)	Reference
Coffee husk	CNCs	20 ± 4	310 ± 160	92	61.8 ± 2.6	[47]
Parchment	CNFs	N/A	N/A	73.4	86.1	[48]
	microfibrils	N/A	N/A	74.5	20.5	[7]
	microfibrils	3	N/A	72	N/A	[49]
Coffee pulp	CNCs	16.03 ± 4.70	N/A	80.5	N/A	[50]
	microfibrils	N/A	N/A	59.63	N/A	[51]
	CNCs	7.9 ± 1.8	77.9 ± 13.6	72	12	[52]
Spent coffee ground	CNCs	17 ± 4	199 ± 27	74.2	10	[53]
	CNFs	25	N/A	72	N/A	[54]
	CNCs	N/A	115	72	7	[55]

## Data Availability

Not applicable.

## Abbreviations

PBAT	poly(butylene adipate-co-terephthalate)
ICO	International Coffee Organization
CNCs	cellulose nanocrystals
CNFs	cellulose nanofibers
TEMPO	2,2,6,6-tetramethylpiperidine-1-oxyl
SEM	scanning electron microscopy
XRD	X-ray diffraction
FTIR	Fourier transform infrared spectroscopy
TGA	thermogravimetric analysis
TEM	transmission electron microscopy
PVA	polyvinyl alcohol
DES	deep eutectic solvent
PLA	poly(lactic acid)
DLS	dynamic light scattering
BDO	1,4-butanediol
AA	adipic acid
PTA	terephthalic acid
HR-TEM	high-resolution transmission electron microscopy
AFM	atomic force microscopy
PCL	polycaprolactone
PHB	poly-3-hydroxybutyrate
PBS	poly(butylene succinate)
PBSA	poly(succinate-co-butylene adipate)
TPS	thermoplastic starch
DMTA	dynamic-mechanical analyses]
DSC	differential scanning calorimetry
DMF	dimethylformamide
TEVS	triethoxyinylsilane
TMPS	trimethoxyphenylsilane
OTR	oxygen permeability
PGMA	poly(glycidyl methacrylate)
ADPM	admicellar polymerization
CTP	hexadecyl pyridium chloride monohydrate
FE-SEM	field emission scanning electron microscopy
